# Frequent structural variations involving programmed death ligands in Epstein-Barr virus-associated lymphomas

**DOI:** 10.1038/s41375-019-0380-5

**Published:** 2019-01-25

**Authors:** Keisuke Kataoka, Hiroaki Miyoshi, Seiji Sakata, Akito Dobashi, Lucile Couronné, Yasunori Kogure, Yasuharu Sato, Kenji Nishida, Yuka Gion, Yuichi Shiraishi, Hiroko Tanaka, Kenichi Chiba, Yosaku Watatani, Nobuyuki Kakiuchi, Yusuke Shiozawa, Tetsuichi Yoshizato, Kenichi Yoshida, Hideki Makishima, Masashi Sanada, Masahiro Onozawa, Takanori Teshima, Yumiko Yoshiki, Tadao Ishida, Kenshi Suzuki, Kazuyuki Shimada, Akihiro Tomita, Motohiro Kato, Yasunori Ota, Koji Izutsu, Ayako Demachi-Okamura, Yoshiki Akatsuka, Satoru Miyano, Tadashi Yoshino, Philippe Gaulard, Olivier Hermine, Kengo Takeuchi, Koichi Ohshima, Seishi Ogawa

**Affiliations:** 1 Department of Pathology and Tumor Biology, Graduate School of Medicine, Kyoto University, Kyoto, Japan; 2 Division of Molecular Oncology, National Cancer Center Research Institute, Tokyo, Japan; 3 Department of Pathology, Kurume University School of Medicine, Kurume, Japan; 4 Pathology Project for Molecular Targets, Cancer Institute, Japanese Foundation for Cancer Research, Tokyo, Japan; 5 Department of Hematology, Necker Hospital, Assistance Publique-Hôpitaux de Paris (APHP), Paris, France; 6 INSERM UMR 1163 and CNRS ERL 8254, Laboratory of Cellular and Molecular Mechanisms of Hematological Disorders and Therapeutical Implications, Imagine Institute, Paris, France; 7 Paris Descartes-Sorbonne Paris Cité University, Paris, France; 8 Department of Pathology, Okayama University Graduate School of Medicine, Dentistry and Pharmaceutical Sciences, Okayama, Japan; 9 Division of Pathophysiology, Okayama University Graduate School of Health Sciences, Okayama, Japan; 10 Laboratory of DNA Information Analysis, Human Genome Center, Institute of Medical Science, The University of Tokyo, Tokyo, Japan; 11 Department of Advanced Diagnosis, Clinical Research Center, Nagoya Medical Center, Nagoya, Japan; 12 Department of Hematology, Faculty of Medicine, Hokkaido University, Sapporo, Japan; 13 Department of Hematology, Japanese Red Cross Medical Center, Tokyo, Japan; 14 Department of Hematology and Oncology, Nagoya University Graduate School of Medicine, Nagoya, Japan; 15 Department of Pediatric Hematology and Oncology Research, National Centre for Child Health and Development, Tokyo, Japan; 16 Department of Pathology, Toranomon Hospital, Tokyo, Japan; 17 Department of Hematology, Toranomon Hospital, Tokyo, Japan; 18 Division of Immunology, Aichi Cancer Center Research Institute, Nagoya, Japan; 19 Department of Hematology, Fujita Health University School of Medicine, Toyoake, Japan; 20 Department of Pathology, Henri-Mondor Hospital, Assistance Publique-Hôpitaux de Paris (AP-HP), Créteil, France; 21 INSERM U955 équipe 9, Institut Mondor de Recherche Biomédicale, Créteil, France; 22 Paris-Est University, Créteil, France

**Keywords:** Cancer genomics, Immunosurveillance, Tumour virus infections

## Abstract

Viral infection induces potent cellular immunity and activated intracellular signaling, which may dictate the driver events involved in immune escape and clonal selection of virus-associated cancers, including Epstein-Barr virus (EBV)-positive lymphomas. Here, we thoroughly interrogated *PD-L1/PD-L2*-involving somatic aberrations in 384 samples from various lymphoma subtypes using high-throughput sequencing, particularly focusing on virus-associated lymphomas. A high frequency of *PD-L1/PD-L2*-involving genetic aberrations was observed in EBV-positive lymphomas [33 (22%) of 148 cases], including extranodal NK/T-cell lymphoma (ENKTL, 23%), aggressive NK-cell leukemia (57%), systemic EBV-positive T-cell lymphoproliferative disorder (17%) as well as EBV-positive diffuse large B-cell lymphoma (DLBCL, 19%) and peripheral T-cell lymphoma-not otherwise specified (15%). Predominantly causing a truncation of the 3′-untranslated region, these alterations represented the most prevalent somatic lesions in ENKTL. By contrast, the frequency was much lower in EBV-negative lymphomas regardless of histology type [12 (5%) of 236 cases]. Besides *PD-L1/PD-L2* alterations, EBV-positive DLBCL exhibited a genetic profile distinct from EBV-negative one, characterized by frequent *TET2* and *DNMT3A* mutations and the paucity of *CD79B*, *MYD88*, *CDKN2A*, and *FAS* alterations. Our findings illustrate unique genetic features of EBV-associated lymphomas, also suggesting a potential role of detecting *PD-L1/PD-L2*-involving lesions for these lymphomas to be effectively targeted by immune checkpoint blockade.

## Introduction

Epstein-Barr virus (EBV) is one of the most prevalent human viruses. The interplay between EBV replication, latency, and immune control can be disrupted, evoking prolonged proliferation of EBV-infected lymphocytes and their malignant transformation [1, 2]. Since its discovery as the first human tumor virus, EBV has been implicated in the development of a wide range of human cancers, including Burkitt lymphoma, EBV-positive T-cell and NK-cell proliferations, and a subset of diffuse large B-cell lymphoma (DLBCL) [1, 2]. The oncogenic potential of EBV-encoded products has been extensively studied; they are known to mimic a variety of cellular factors involved in cell growth, transcription, and apoptosis, to usurp control of the pathways regulating diverse homeostatic cellular functions. However, despite this oncogenic potential, the virus induces lymphoma only in a fraction of EBV-infected people, generally after a long latency period. Thus, EBV is thought to require somatic alterations in the cellular genome to cause lymphoma, whose impacts on the development of EBV-positive lymphomas have not fully been investigated, even though they might substantially differ from those involved in the development of EBV-negative lymphomas.

Immune checkpoint blockade using anti-PD-1/PD-L1 antibodies is a highly promising therapy that can induce a durable anti-tumor response and a long-term remission in a wide variety of cancer types [3–5]. In particular, an excellent response to anti-PD-1 antibodies has been demonstrated for advanced cases with classical Hodgkin lymphoma (cHL), a defining feature of which is frequent copy number gains or amplifications involving *PD-L1* and/or *PD-L2*, suggesting a close link between *PD-L1/PD-L2* genetic alterations and the therapeutic response to these agents [6–8]. In addition to cHL, several subtypes of B-cell lymphomas are shown to have structural variations (SVs) involving PD-1 ligands, such as chromosomal translocation causing promoter replacement [9–12]. Moreover, recently we have reported frequent SVs disrupting the 3′-untranslated region (UTR) of *PD-L1* in multiple cancers, especially in adult T-cell leukemia/lymphoma (ATL) and DLBCL [13, 14]. These observations point to a potential of diverse *PD-L1/PD-L2* somatic alterations as a key driver of lymphomagenesis. However, the comprehensive landscape of these alterations in many subtypes of non-Hodgkin lymphomas (NHLs) remains elusive.

In this study, we first interrogated *PD-L1/PD-L2-*involving genetic aberrations in various lymphoma subtypes and found their frequent involvement in EBV-associated lymphomas. We further investigated different underlying genetic mechanisms between EBV-positive and -negative tumors.

## Materials and methods

### Patient samples

A total of 384 lymphoma specimens of different histology types, including 337 samples from Japan and 47 samples from France [TENOMIC consortium of the Lymphoma Study Association (LYSA) group], were enrolled in this study according to the protocols approved by the Institutional Review Boards (IRB, Supplemental Table S1). All samples were collected from patients with informed consent, except for already-collected, anonymized samples whose use was permitted by the IRB. Pathological diagnosis was based on the 2008 World Health Organization (WHO) classification [15], except for EBV-positive DLBCL, for which the restriction to elderly patients was not applied according to the recent revision of the WHO classification [16]. This study was approved by the institutional ethics committees of the Graduate School of Medicine, Kyoto University and other participating institutes.

### Detection of *PD-L1/PD-L2*-involving genetic alterations

SVs and focal copy number alterations (CNAs) affecting PD-1 ligands were explored using targeted-capture sequencing with a custom SureSelect library (Agilent Technologies, Santa Clara, CA, USA) designed for capturing the entire sequences of the *PD-L1* and *PD-L2* genes, including their exons, introns, and 5′- and 3′-UTRs (Supplemental Figure S1). Sequencing data were obtained using the Illumina HiSeq 2500 platform with a standard 125-bp paired-end read protocol. SVs and focal CNAs were detected using the Genomon pipeline (https://github.com/Genomon-Project/) and the CNACS algorithm, respectively, as previously described [13, 17, 18]. Putative SVs were manually curated and further filtered by removing those (i) with Fisher’s exact *P*-value >0.1; (ii) with <4 supporting reads in tumor; or (iii) present in any of control samples. SV breakpoints were visually inspected using Integrative Genomics Viewer (IGV). Candidate focal CNAs (< 20 Mb in length) were also manually reviewed and further filtered by removing those with <3 probes.

### Detection of EBV genome

For the detection of EBV sequence, after sequencing reads were mapped to the EBV genome (NC_007605), the number of the EBV-aligned reads in proper pairs were enumerated and divided by the number of total reads uniquely mapped to the human reference genome (GRCh37). Then, the obtained ratio was evaluated for the cut-off value of 0.00015%, which was determined so that all EBV-positive cases with DLBCL and extranodal NK/T-cell lymphoma (ENKTL) confirmed by in situ hybridization (ISH) for EBV-encoded small RNA (EBER), LMP1 immunohistochemistry (IHC), or Southern blot were included, while minimizing EBV-negative cases. RNA sequencing (RNA-seq) data from the Cancer Genome Atlas (TCGA) cohort were analyzed in a similar manner, where the detection of EBV reads was considered as positive. Southern blot for EBV genome was performed according to a standard procedure.

### Whole-exome analysis of ENKTL

Whole-exome sequencing data for ENKTL (accession number SRP057085) were obtained from the National Center for Biotechnology Information Sequence Read Archive and applied for the detection of somatic alterations using the Genomon pipeline, as previously described [18]. Candidate mutations with (i) Fisher’s exact *P*-value ≤0.01; (ii) ≥4 variant reads in tumor; and (iii) allele frequency in tumor ≥0.025 were adopted and filtered by excluding (i) synonymous single nucleotide variants (SNVs); (ii) known variants listed in the 1000 Genomes Project (October 2014 release), NCBI dbSNP build 131, National Heart, Lung, and Blood Institute (NHLBI) Exome Sequencing Project (ESP) 6500, the Human Genome Variation Database (version 2.0), or our in-house single nucleotide polymorphism (SNP) database, unless they were listed in the COSMIC database (v70). SVs and focal CNAs were analyzed in the same manner as described above.

### Targeted-capture sequencing of EBV-negative and -positive DLBCLs

Targeted-capture sequencing was performed using a custom SureSelect library (Agilent Technologies) for 140 lymphoma-associated genes (Supplemental Table S2), as previously described [18, 19]. Additional probes for 1999 SNPs were designed to calculate genomic copy numbers [17]. Somatic mutations with (i) ≥5 variant reads in tumor; and (ii) allele frequency in tumor ≥0.025 were adopted and filtered by removing (i) synonymous SNVs; (ii) known variants listed in SNP databases (as described above); (iii) variants only present in unidirectional reads; (iv) variants occurring in repetitive genomic regions. Candidate mutations were further filtered by removing missense SNVs with allele frequency of 0.4–0.6. Finally, mapping errors were removed by visual inspection with IGV. SVs and focal CNAs were analyzed in the same manner as described above.

### RNA-seq and expression microarray analysis

RNA-seq data for endemic Burkitt lymphoma [20] (SRP062178), anaplastic large cell lymphoma [21] (ALCL, SRP044708), and cutaneous T-cell lymphoma [22] (CTCL, SRP058948) were obtained from the National Center for Biotechnology Information Sequence Read Archive. RNA-seq data for peripheral T-cell lymphoma [23] (PTCL, phs000689.v1.p1) and primary mediastinal B-cell lymphoma [24] (PMBCL, EGAD00001000692) were obtained through Database of Genotypes and Phenotypes and European Genome-phenome Archive, respectively. Microarray data for normal human cells were obtained from HemaExplorer [25]. For RNA-seq data, expression quantification and detection of fusion transcripts were performed using the Genomon pipeline, as previously described [13, 18].

### Analysis of TCGA data sets

As previously reported, we analyzed 10,210 TCGA samples from 33 cancer types, for which RNA-seq data were available from the Cancer Genomic Hub (https://cghub.ucsc.edu), to search for 3′-UTR-disrupted *PD-L1* and *PD-L2* transcripts [13]. To evaluate the copy number of *PD-L1* and *PD-L2*, the level 3 segmented copy number data (Affymetrix Genome-Wide Human SNP Array 6.0) were downloaded from the TCGA data portal (https://tcga-data.nci.nih.gov) for DLBCL and stomach adenocarcinoma samples.

### Immunohistochemical analysis

IHC for PD-L1 was performed on formalin-fixed paraffin-embedded tissue sections using antibodies directed against the N-terminal (E1J2J, Cell Signaling Technology, Beverly, MA, USA) and C-terminal (SP142, Spring Bioscience, Fremont, CA, USA) domains of PD-L1. IHC for Ki-67 was performed using a mouse monoclonal antibody (MIB-1, Dako, Tokyo, Japan). The antigen–antibody complexes were visualized with Histofine Simple Stain MAX PO (Nichirei Bioscience, Tokyo, Japan). Some sections were double-stained with PD-L1 (E1J2J) and CD3, using a mouse monoclonal antibody (F7.2.38, Dako) with PolyView IHC reagent (mouse-AP, Enzo Life Sciences, Farmingdale, NY, USA) (Supplemental Figure S2). A tumor sample was considered positive for PD-L1 expression, when >5% of tumor cells were stained, where PD-L1 expression in tumor cells was discriminated from that in immune cells on the basis of cell morphology and/or cytoplasmic CD3 expression. LMP1 expression was detected using a mouse monoclonal antibody (CS1-4, Dako) with the EnVision system (Dako). EBER-ISH was performed on the Leica Bond-III Automatic Stainer (Leica Microsystems, Wetzlar, Germany) using Bond Ready-to-Use ISH EBER Probe and Bond Ready-to-Use Anti-Fluorescein Antibody (Leica Biosystems, Wetzlar, Germany).

### Fluorescent in situ hybridization (FISH)

FISH analyses were performed on unstained slides with bacterial artificial chromosome (BAC) clone-derived DNA probes for the 5′- and 3′-parts of *PD-L1* locus. The hybridized slides were then counterstained with 4′,6-diamidino-2-phenylindole (DAPI) and observed with a fluorescence microscope BX51 (Olympus, Tokyo, Japan).

### CRISPR-mediated gene targeting

CRISPR-mediated gene editing was used to generate *PD-L1* and *PD-L2* 3′-UTR disruptions, as previously described [13]. Briefly, human *PD-L1* and *PD-L2* 3′-UTR single guide RNA (sgRNA) targeted sites were designed manually and checked in silico. The pSpCas9(BB)-2A-GFP (pX458) vector expressing Cas9 (Addgene plasmid 48138) was digested with BbsI and ligated to the annealed sgRNA oligonucleotides. HEK293T (obtained from the RIKEN Cell Bank) and T2 (a gift from H. Kawamoto, Kyoto University) cells were transfected with indicated vectors using X-tremeGENE 9 DNA Transfection Reagent (Roche, Basel, Switzerland) and the Neon transfection system (Thermo Fisher Scientific, Waltham, MA, USA), respectively. To validate CRISPR/Cas9-mediated disruption of *PD-L2* 3′-UTR, genomic PCR flanking the breakpoint region was performed. The sgRNA and primer sequences are listed in Supplemental Tables S3, S4. Cell lines were authenticated by the providers and routinely tested for mycoplasma infection.

### Flow cytometric analysis

To detect surface PD-L1 or PD-L2 expression, cells were stained with allophycocyanin (APC)-conjugated anti-CD274 (29E.2A3) and anti-CD273 (24F.10C12) antibodies (BioLegend, San Diego, CA, USA), and analyzed on FACS LSR Fortessa (BD Biosciences, San Jose, CA, USA). The data analyses were performed with FlowJo software (TreeStar, Ashland, OR, USA).

### Statistical analysis

Statistical analyses were performed with R3.4.2 software (The R Foundation for Statistical Computing). For functional assays, statistical significance was assessed by Student’s two-tailed *t*-test with a Welch’s correction. The molecular features between EBV-negative and -positive tumors were compared using the Fisher’s exact test, Cochran–Armitage trend test, or Brunner–Munzel test. *P*-values were considered statistically significant at <0.05. In box plots, the center line and lower and upper hinges correspond to the median, and the first and third quartiles (25 and 75 percentiles), respectively. The upper and lower whiskers extend from the upper and lower hinges to the largest or smallest values no further than 1.5× inter-quartile range from the hinges.

### Code availability

The code for the Genomon pipeline is available at https://github.com/Genomon-Project/.

## Results

### Analysis of *PD-L1/PD-L2* genetic alterations in a variety of B- and T/NK-cell lymphomas

**Fig. 1:**
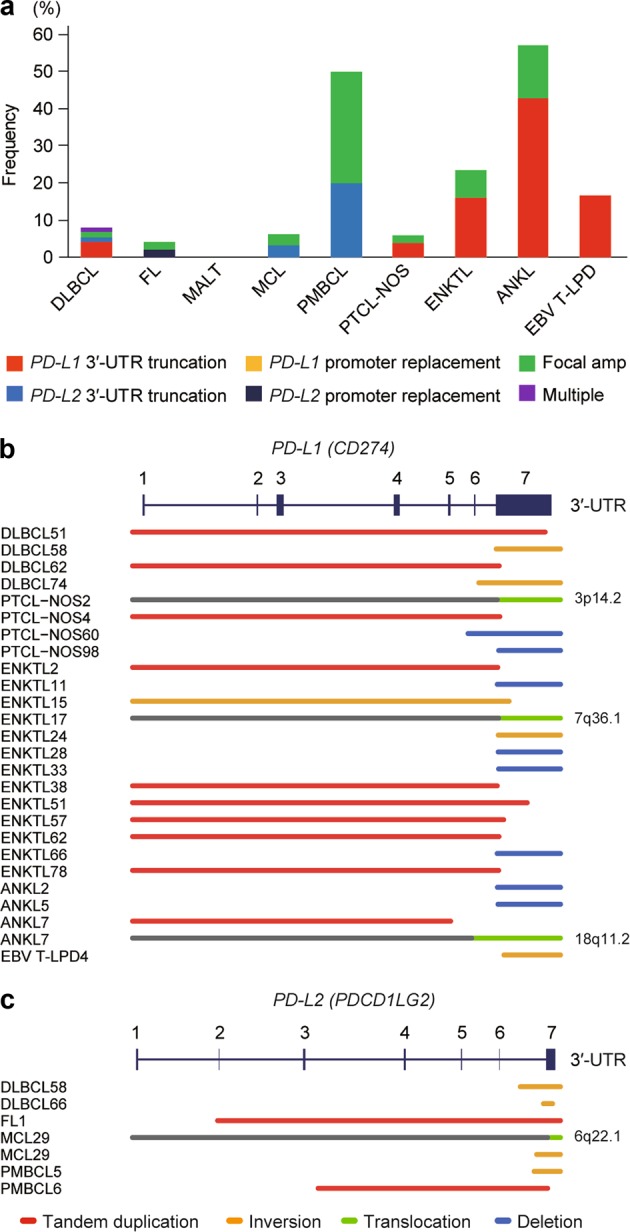
Genetic alterations involving PD-1 ligands in various subtypes of lymphomas. **a** Frequency of genetic alterations involving *PD-L1* and/or *PD-L2* in each lymphoma subtype. Type of alterations is indicated by color. Cases harboring both SV and focal CNA affecting *PD-L1* and/or *PD-L2* are combined into the corresponding SV group. Multiple represents cases harboring both *PD-L1* and *PD-L2* SVs. DLBCL, diffuse large B-cell lymphoma; FL, follicular lymphoma; MALT, mucosa-associated lymphoid tissue lymphoma; MCL, mantle cell lymphoma; PMBCL, primary mediastinal B-cell lymphoma; PTCL-NOS, peripheral T-cell lymphoma-not otherwise specified; ENKTL, extranodal NK/T-cell lymphoma; ANKL, aggressive NK-cell leukemia; EBV T-LPD, Systemic EBV-positive T-cell lymphoproliferative disorder; Amp, amplification. **b**, **c** Different types of SVs affecting *PD-L1* (**b**) and *PD-L2* (**c**) are shown by indicated colors

*PD-L1*/*PD-L2*-involving genetic abnormalities were interrogated using targeted-capture sequencing in a total of 384 samples from different lymphoma subtypes, including DLBCL (*n* = 75), follicular lymphoma (FL, *n* = 49), mucosa-associated lymphoid tissue lymphoma (MALT, *n* = 20), mantle cell lymphoma (MCL, *n* = 32), PMBCL (*n* = 10), PTCL-not otherwise specified (PTCL-NOS, *n* = 104), and EBV-positive T-cell and NK-cell proliferations (*n* = 94) (Supplemental Table S1). Overall, SVs affecting *PD-L1* and *PD-L2* were detected in 25 (7%) and 6 (2%) samples, respectively, while focal gains or amplifications involving these genes were found in 30 (8%) samples (Supplemental Figures S1 and S3 and Supplemental Table S5). These alterations were observed in both B-cell and T/NK-cell lymphomas, with varying frequency (0-57%) depending on histology (Fig. 1a). Regardless of SV types involved (deletion, inversion, tandem duplication, and translocation), most of these SVs resulted in 3′-UTR truncation of the corresponding genes (Fig. 1b, c). Although reported in previous publications [9–12], translocations of the *PD-L1* or *PD-L2* promoter to an ectopic regulatory locus was rarely observed. Except for Burkitt lymphoma [20] (0 of 20 samples), similar results were obtained from the analysis of publicly available transcriptome sequencing data of PTCL [23] (1 of 35), ALCL [21] (1 of 23), CTCL [22] (1 of 13), and PMBCL [24] (3 of 6), where an marked increase in *PD-L1* or *PD-L2* mRNA expression was observed in most cases with *PD-L1*- or *PD-L2*-involving aberrant transcripts (Supplemental Figure S4A–C and Supplemental Table S6). In addition, a PTCL-NOS case with a *PD-L1* 3′-UTR truncation showed strong PD-L1 expression in IHC (Supplementary Figure S3D and Supplemental Table S7). Of interest were those cases in which multiple SVs involving *PD-L1* and/or *PD-L2* were observed in a single tumor (DLBCL58, MCL29, and ANKL7), underscoring the importance of the deregulated expression of these PD-1 ligands in clonal selection and expansion of these tumors.

### *PD-L2* SV is a characteristic genetic alteration of B-cell lymphomas

**Fig. 2:**
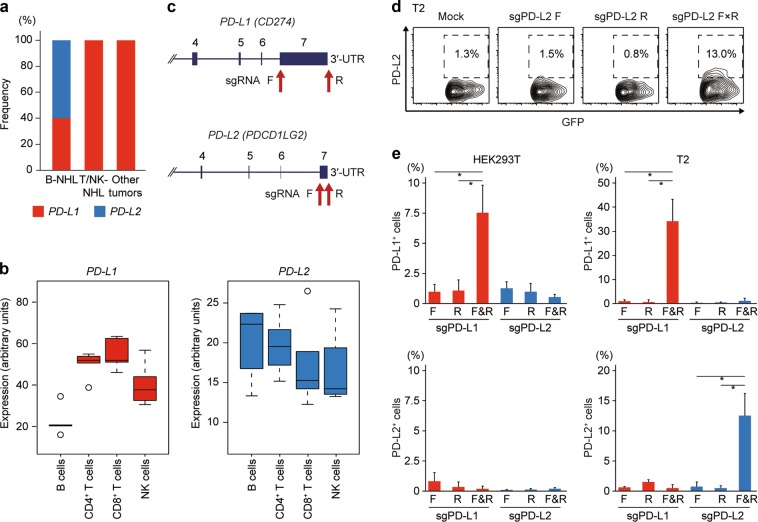
CRISPR/Cas9-mediated 3′-UTR disruption induces PD-L2 overexpression. **a** Proportion of *PD-L1* and *PD-L2* SVs in B-NHL and T/NK-NHL as well as other tumors. **b** *PD-L1* and *PD-L2* mRNA expression in normal human B, CD4^+^ T, CD8^+^ T, and NK cells. Expression microarray data were obtained from HemaExplorer [25]. **c** Positions of targeting sgRNAs used for CRISPR/Cas9-mediated deletions and inversions of *PD-L1* and *PD-L2* 3′-UTR. **d,** **e** Representative plots (**d**) and frequencies (**e**) of PD-L1^+^ (top) or PD-L2^+^ (bottom) cells in green fluorescent protein (GFP)^+^ fraction by flow cytometry in HEK293T (**e**, left) or T2 (**d**, **e**, right) cells transfected with Cas9 and no, single, or pairwise sgRNAs targeting *PD-L1* or *PD-L2* 3′-UTR (n = 3 biological replicates). Data represent means ± s.d. *P < 0.05, Welch’s *t-*test

In contrast to *PD-L1*-involving SVs, which were detected in both B- and T/NK-cell lymphomas, those involving *PD-L2* were found exclusively in B-cell lymphomas (Figs 1a and 2a). Similarly, while *PD-L1* SVs are present in a variety of solid cancers [13], no *PD-L2*-involving SVs were identified among 10,162 cancer samples from 32 tumor panels, for which RNA-seq data were available from TCGA. These results suggest that *PD-L1* is affected in a broad spectrum of human malignancies, whereas *PD-L2* SVs are largely restricted to B-cell lymphomas, possibly reflecting the expression pattern of PD-1 ligands (Fig. 2b). Based on these findings, we assessed whether disruption of *PD-L2* 3′-UTR could induce PD-L2 overexpression using CRISPR/Cas9-mediated gene editing, as was the case with enhanced PD-L1 expression through disruption of its 3′-UTR (Fig. 2c). Although disruption of *PD-L2* 3′-UTR resulted in a significant elevation of PD-L2 expression in T2 human B and T lymphoblast hybrid cell line (Fig. 2d, e and Supplemental Figure S5), it did not affect PD-L2 expression in HEK293T cells, confirming the restricted relevance of 3′-UTR-mediated regulation of PD-L2 expression to B-cell lineage.

### Frequent *PD-L1* genetic alterations in EBV-positive T-cell and NK-cell proliferations

**Fig. 3:**
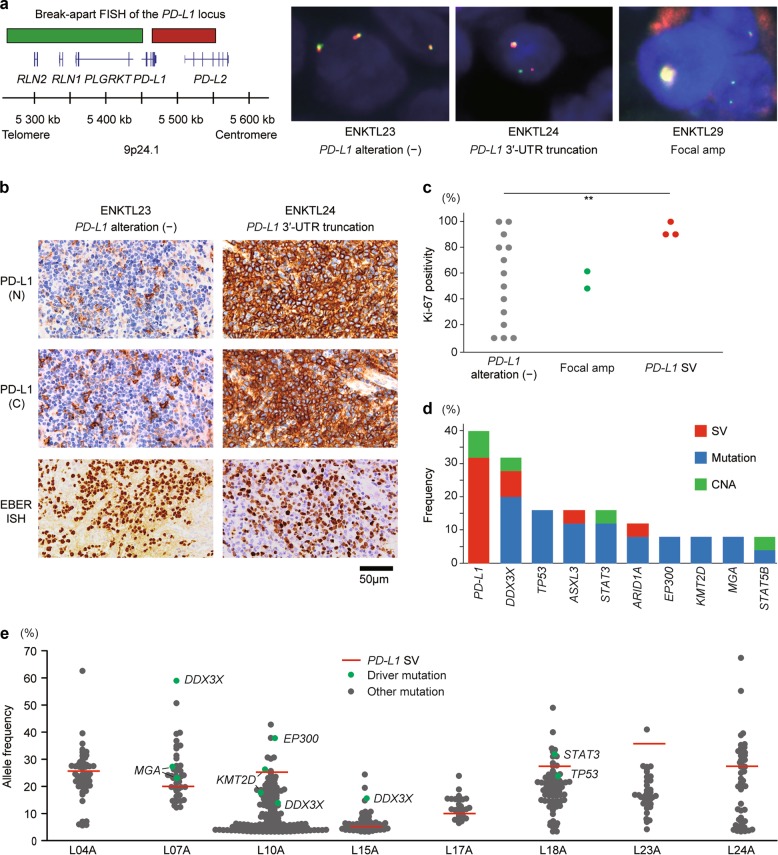
ENKTL harbors frequent *PD-L1* SVs, leading to its overexpression. **a** FISH analysis showing *PD-L1* break-apart (middle) and cluster formation (right) in ENKTL cells. Design of the break-apart assay using BAC probes recognizing the 5′-part (green) and 3′-part (red) of *PD-L1* loci is shown. Amp, amplification. **b** PD-L1 IHC (top and middle) and EBER-ISH (bottom) of ENKTL cases with or without *PD-L1* genetic alterations. Antibodies specifically detecting N-terminal (top) and C-terminal (middle) domains of PD-L1 were used. **c** Percentage of Ki-67^+^ cells in tumor cell fraction in ENKTL cases with or without *PD-L1* genetic alterations. Each dot represents a single case. **P < 0.005, Brunner–Munzel test. A summary of the results is shown in Table 1. **d** Frequently altered genes identified by whole-exome sequencing for 25 ENKTL cases [28]. Type of somatic alterations is indicated by color. **e** Hierarchy of somatic mutations and *PD-L1* SVs is shown with their allele frequencies in 8 ENKTL cases analyzed by whole-exome sequencing. Driver mutations are shown in green, and *PD-L1* SVs are shown in red

Consistent with previous reports [10, 11], *PD-L1/PD-L2*-involving lesions were frequently observed in PMBCL, accounting for a half of the cases (Fig. 1a). Notably, mainly consisting of its 3′-UTR truncations, *PD-L1*-involving abnormalities were prevalently found in EBV-positive T-cell and NK-cell proliferations (17–57%), including ENKTL (19 of 81 samples), aggressive NK-cell leukemia (ANKL, 4 of 7), and systemic EBV-positive T-cell lymphoproliferative disorder (EBV T-LPD, 1 of 6) (Fig. 1a, b). Among these, the most common entity is ENKTL, which is an aggressive neoplasm with a predilection for Asian and South American populations [26]. These *PD-L1*-involving SVs and focal CNAs were confirmed using multi-color FISH in all ENKTL cases by way of positive break-apart and clustered signals, respectively (Fig. 3a and Table 1). In IHC, PD-L1 expression was detected in 6 of 19 ENKTL cases examined, where almost all tumor cells were positive for PD-L1 (Fig. 3b and Table 1). Among them, 4 cases had *PD-L1*-involving genetic lesions, while all ENKTL cases having *PD-L1*-involving genetic lesions were positive for PD-L1 expression in IHC, except for one case with a focal *PD-L1* amplification. These findings suggest that *PD-L1*-involving genetic abnormalities are the major cause of PD-L1 overexpression in ENKTL, although other mechanisms, such as induction by EBV-encoded products, might operate, particularly in those cases with positive IHC but lacking detectable *PD-L1* genetic lesions [27]. Additionally, samples with *PD-L1* 3′-UTR truncations showed a higher percentage of Ki-67 positive cells, compared with *PD-L1* SV-negative ones, suggesting a possible intrinsic role for PD-L1 in regulating cellular growth (Fig. 3c and Table 1). To determine the relative contribution of *PD-L1*-involving genetic alterations in NK/T-cell lymphomagenesis, we analyzed published exome data of 25 ENKTL cases for other somatic events than *PD-L1* abnormalities [28]. In accordance with a previous report [28], the most frequently mutated genes included *DDX3X* and *TP53* (Fig. 3d). Moreover, we found that these common drivers were also affected by SVs and CNAs, which were overlooked in the previous study. Nevertheless, the most common genetic alterations in ENKTL were represented by those involving *PD-L1*, which were found in as many as 10 (40%) of ENKTL cases (Fig. 3d, Supplemental Figure S6, and Supplemental Table S8), even though their frequency is likely to be substantially underestimated because of the lack of coverage for intronic and UTR sequences in the exome data to capture the relevant SVs. Analysis of the clonal structure of tumor cells showed that *PD-L1* 3′-UTR truncations were present in major clones in most cases (Fig. 3e), suggesting that *PD-L1* genetic alterations may represent a relatively early event in tumor development.

**Table 1:** *PD-L1* genetic alterations, its expression, and EBV status in ENKTL

Case	*PD-L1* SV	*PD-L1* CNA	*PD-L1* FISH	PD-L1 IHC (N)	PD-L1 IHC (C)	EBER-ISH	LMP1 IHC	Ki-67
ENKTL10	−	−	−	−	−	+	−	30%
ENKTL11	3′-UTR truncation	Focal amp	NE	NE	NE	+	−	NE
ENKTL12	−	−	−	−	−	+	+	80%
ENKTL13	−	−	−	−	−	+	−	100%
ENKTL14	−	−	−	−	−	+	−	10%
ENKTL15	3′-UTR truncation	Focal amp	Cluster formation	+	+	−	+	90%
ENKTL16	−	−	−	−	−	+	+	60%
ENKTL17	3′-UTR truncation	−	Split signal	+	+	+	+	100%
ENKTL18	−	−	−	−	−	−	+	40%
ENKTL19	−	−	NE	NE	NE	+	−	NE
ENKTL20	−	−	−	−	−	+	−	100%
ENKTL21	−	−	−	+	+	+	+	50%
ENKTL22	−	−	−	+	+	+	+	70%
ENKTL23	−	−	−	−	−	+	−	80%
ENKTL24	3′-UTR truncation	Focal amp	Split signal	+	+	+	+	90%
ENKTL25	−	Focal amp	Cluster formation	−	−	+	−	60%
ENKTL26	−	−	−	−	−	+	−	10%
ENKTL27	−	−	−	−	−	−	+	20%
ENKTL28	3′-UTR truncation	−	NE	NE	NE	+	−	NE
ENKTL29	−	Focal amp	Cluster formation	+	+	−	+	50%
ENKTL30	−	−	−	−	−	+	−	90%
ENKTL31	−	−	−	−	−	+	−	10%

Results of targeted-capture sequencing (*PD-L1* SV and *PD-L1* CNA), *PD-L1* FISH, IHC for PD-L1 (with the N-terminal or C-terminal antibody), LMP1, and Ki-67, and EBER-ISH for 22 ENKTL cases are shown

*FISH* fluorescent in situ hybridization, *IHC* immunohistochemistry, *EBER-ISH* in situ hybridization for EBV-encoded small RNA, *NE* not evaluable

### Association of EBV infection with genetic alterations of PD-1 ligands

**Fig. 4:**
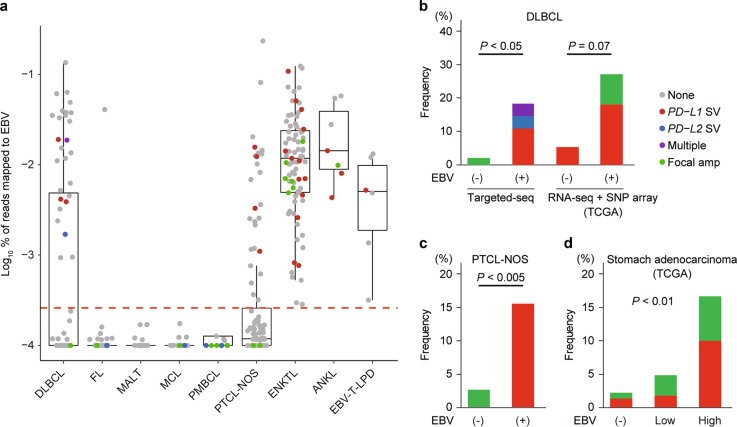
*PD-L1/PD-L2* genetic alterations associated with EBV infection. **a** Proportion of EBV DNA reads to total mapped reads according to lymphoma subtype (offset, 0.0001). Each dot represents a single case. Red line indicates the cut-off value (0.00015%) for EBV positivity. Type of genetic alterations involving PD-1 ligands is indicated by color. Multiple represents cases harboring both *PD-L1* and *PD-L2* SVs. **b**–**d** Frequency of genetic alterations involving *PD-L1/PD-L2* according to EBV status in DLBCL (**b**), PTCL-NOS (**c**), and stomach adenocarcinoma (**d**). Fisher’s exact test is used for DLBCL and PTCL-NOS, and Cochran–Armitage trend test is applied to stomach adenocarcinoma. EBV high group corresponds to the EBV-positive group in the TCGA classification of stomach adenocarcinoma [38]

A high frequency of *PD-L1* SVs was previously reported in a unique form of HTLV-1-mediated PTCL, ATL [13, 14], raising a possibility that genetic defects involving PD-1 ligands play a pivotal role in immune escape of virus-related tumors. In this point of view, it would be of interest to see the frequency of *PD-L1/PD-L2*-involving abnormalities among EBV-associated lymphomas and stomach adenocarcinoma, another EBV-related cancer. Among 75 DLBCL cases in our cohort, 27 cases (DLBCL49-75) were positive for EBV by Southern blot and/or targeted-capture sequencing, and thus considered as EBV-positive DLBCL (Fig. 4a). We observed a significantly higher frequency of *PD-L1*/*PD-L2*-involving genetic aberrations in EBV-positive DLBCL (5 of 27) than in EBV-negative DLBCL (1 of 48) (*P* < 0.05) (Fig. 4a, b). The observation was confirmed by examining DLBCL cases in the TCGA cohort (27% vs. 5%, *P* = 0.07) (Fig. 4b). Similar trends were also seen for PTCL-NOS (Fig. 4a, c), as well as stomach adenocarcinoma from the TCGA cohort (Fig. 4d). These results suggest a strong relationship between EBV infection and *PD-L1/PD-L2*-involving genetic abnormalities.

### Different underlying genetic mechanisms between EBV-negative and -positive DLBCLs

**Fig. 5:**
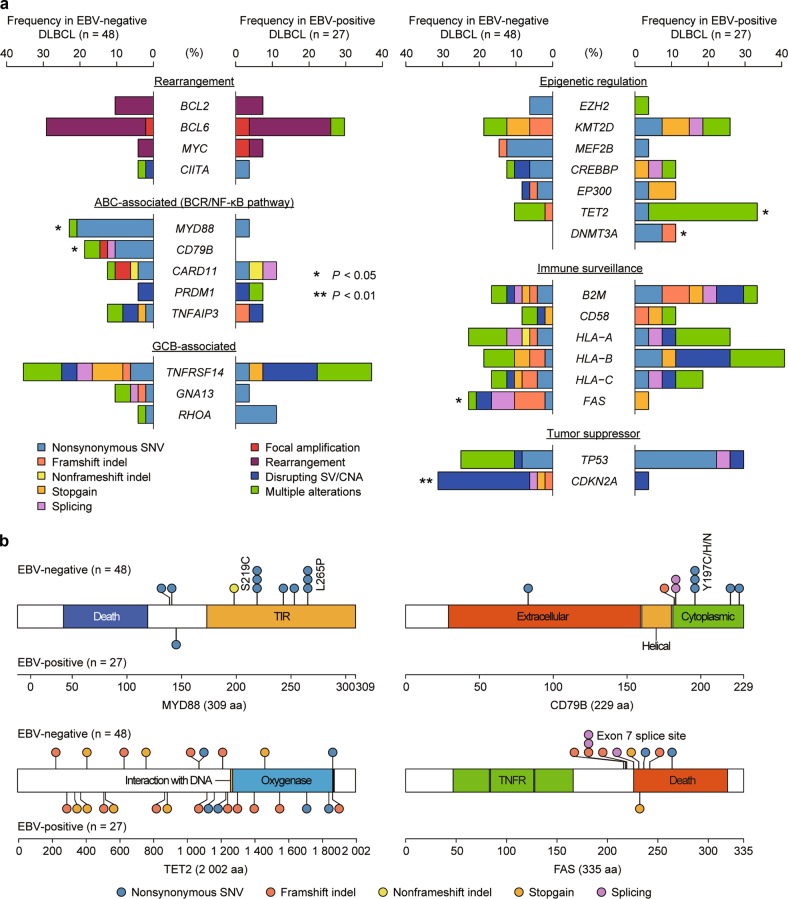
Genetic differences between EBV-negative and -positive DLBCLs. **a** Frequency and type of somatic alterations identified by targeted-capture sequencing for lymphoma-associated genes in 48 EBV-negative and 27 EBV-positive DLBCL cases. Type of alterations is indicated by color. BCR, B-cell receptor; GCB, germinal center B-cell. *P < 0.05, **P < 0.01, Fisher’s exact test. **b** Distribution of somatic mutations encoded in *MYD88* (NM_002468), *CD79B* (NM_000626), *TET2* (NM_001127208), and *FAS* (NM_000043) detected in 48 EBV-negative and 27 EBV-positive DLBCL cases. The protein domains were obtained from the NCBI Gene database (https://www.ncbi.nlm.nih.gov/gene/) and the UniProt database (https://www.uniprot.org/)

Based on these findings, we hypothesized that EBV-positive tumors may constitute a genetically distinct neoplasm from the EBV-negative counterpart. Thus, we compared frequencies of major driver alterations implicated in lymphomagenesis between EBV-positive and -negative DLBCLs (Supplemental Tables S9–S11). Although the numbers of mutations and CNAs were comparable between EBV-negative and -positive DLBCLs, EBV-positive DLBCLs had a larger number of SVs than EBV-negative tumors (Supplemental Figure S7A). The frequency and type of somatic alterations observed in our cohort were quite similar to previous studies [29, 30] (Supplemental Figure S7B). Conspicuously, compared to EBV-negative DLBCLs, EBV-positive DLBCLs showed substantially higher frequencies of *TET2* and *DNMT3A* mutations, while showing very low frequencies of *CD79B* and *MYD88* mutations as well as *CDKN2A* loss-of-function alterations (Fig. 5a, b). In addition, despite the common occurrence of *PD-L1/PD-L2* somatic alterations, EBV-positive tumors were largely devoid of *FAS* mutations and deletions, which are also thought to be involved in escape from immune surveillance by T cells [31]. There were no differences in genetic alterations between EBV-positive DLBCLs with and without *PD-L1/PD-L2* genetic alterations (Supplemental Figure S7C). Taken together, these observations suggest the presence of distinct genetic mechanisms for the pathogenesis of EBV-positive and -negative DLBCLs.

## Discussion

We have revealed a high prevalence of *PD-L1/PD-L2*-involving SVs and/or focal amplifications and accompanying overexpression of the corresponding PD-1 ligand in a wide variety of lymphomas, suggesting a critical role of evasion from cellular immunity in the development of these lymphomas. Of particular interest in this respect is especially high frequencies of these abnormalities in EBV-associated lymphomas, which were detected in as many as 22% of these lymphomas. Affecting more than 90% of adult individuals, EBV infection represents one of the most prevalent viral infections among human populations, where it induces potent cellular immunity [1, 2]. In fact, up to 5% of cytotoxic T cells in our body are devoted to eliminate EBV-infected cells [1]. Intact immunity to EBV is essential to self-terminate infectious mononucleosis or prevent other lymphoproliferative diseases associated with transplantation or other immunocompromised status [1, 2]. Thus, evasion from anti-viral immunity is thought to be a critical step for EBV-infected B or T/NK cells to achieve neoplastic growth, except for Burkitt lymphoma, which exhibits a restrictive pattern of expression of latent encoded proteins (type 1 latency) [1, 2]. In this context, EBV-infected cells that acquire *PD-L1/PD-L2*-involving alterations are thought to effectively evade anti-EBV immune surveillance to be clonally selected for further neoplastic outgrowth, which in turn points to a possibility that checkpoint blockade targeting the PD-1/PD-L1 axis might provide effective therapeutics against EBV-related lymphomas otherwise associated with a dismal prognosis with conventional chemotherapy [26, 32]. In agreement with this hypothesis is a recent report describing a remarkable activity of anti-PD-1 antibody against patients with relapsed or refractory ENKTL [33]. Therefore, it would be an emerging question whether or not detection of *PD-L1/PD-L2*-involving alterations helps identify those lymphomas in which an excellent response to PD-L1/PD-L2 blockade is expected, even though the overall efficacy may not be remarkable [34].

Oncogenic viral infections are closely related with the development of various cancers, such as hepatitis B- and C-virus-associated hepatocellular carcinoma, human papilloma virus-associated head and neck cancers, polyomavirus-associated Merkel cell carcinoma, and HTLV-1-associated ATL. In contrast to somatic mutations or SVs of the tumor genome, which can generate a limited number of antigenic peptides for T cell recognition, the entire protein product expressed from viral genes represents non-self, thereby conferring strong neoantigenecity [3]. Therefore, a deregulated PD-1 and PD-L1 axis might be relevant to immune evasion in virus-associated cancers, and thus the presence of the causative virus could represent a predictive biomarker for response to immune checkpoint blockade. In accordance with this theory, substantial efficacy of anti-PD-1 therapy among several virus-related tumors have been noted, although further studies are warranted [3].

Another finding of significant importance is a distinct pattern of somatic alterations in EBV-positive DLBCL, providing new insight into the genetic basis of virus-induced lymphomas. EBV gene products, such as LMP1, have been reported to strongly activate NF-κB pathway in a ligand-independent manner, and hinder p16 (encoded by *CDKN2A*) -Rb pathway, which can explain the absence of *CD79B*, *MYD88*, and *CDKN2A* alterations in EBV-positive DLBCL, although EBV-positive DLBCL is characterized by an activated B-cell (ABC) immunophenotype and prominent NF-κB activation [32, 35]. Frequent *TET2* and *DNMT3A* mutations in EBV-positive DLBCL indicate the possible involvement of deregulated DNA methylation and demethylation processes in this disease [36]. In addition to *PD-L1* 3′-UTR truncation, which is associated with Ki-67 positivity in ENKTL, *TET2* disruption is also reported to cause aberrant proliferation in an antigen receptor-dependent manner [37]. These alterations can contribute to tumor growth, which may explain the paucity of mutations in B-cell receptor (BCR)/NF-κB pathway-associated molecules. Furthermore, an inverse relationship of *PD-L1/PD-L2* and *FAS* alterations suggests different roles of these molecules in escape from T-cell mediated immune surveillance against virally infected cells.

Taken together, our findings contribute to understand the pathogenesis of EBV-associated lymphomas and exploit a new diagnostic strategy to identify patients who are likely to respond to PD-1/PD-L1 blockade therapy by detection of EBV and/or *PD-L1/PD-L2* genetic alterations in NHLs.

## Supplementary information

Supplemental data

Supplemental tables
