# Supplementary material for: Frequent structural variations involving programmed death ligands in Epstein-Barr virus-associated lymphomas
Source: Leukemia. 2019 Jan 25;33(7):1687–99. doi: 10.1038/s41375-019-0380-5 (PMC6755969; doi:10.1038/s41375-019-0380-5)
Supplement: Supplementary file 1 — Supplemental data [file 41375_2019_380_MOESM1_ESM.docx]

## Supplemental data

###
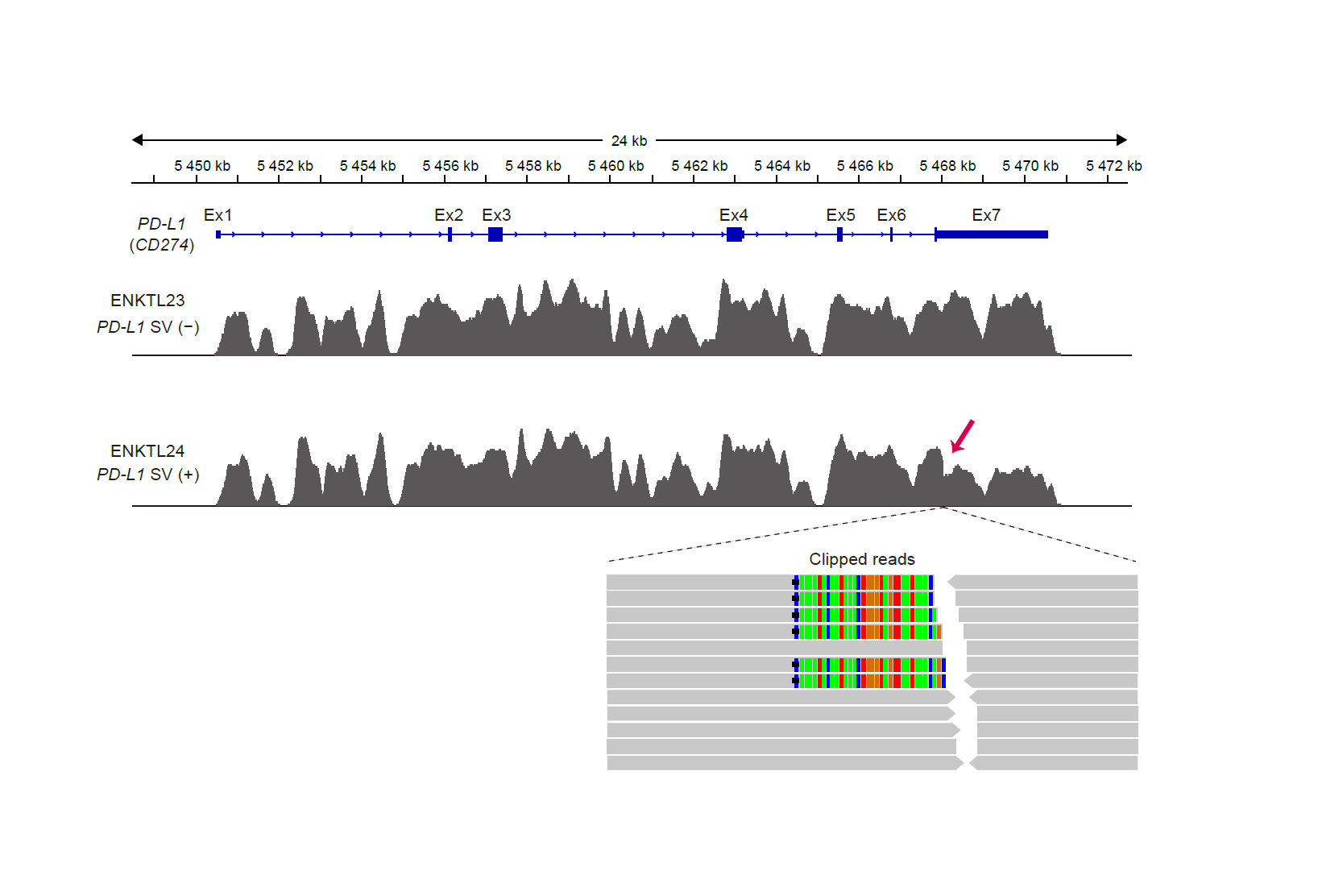
Figure S1 Detection of *PD-L1* SVs by targeted-capture sequencing.

Targeted-capture sequencing data are visualized by IGV for representative ENKTL samples with or without *PD-L1* SVs. Misaligned bases in soft-clipped reads are colored.

###
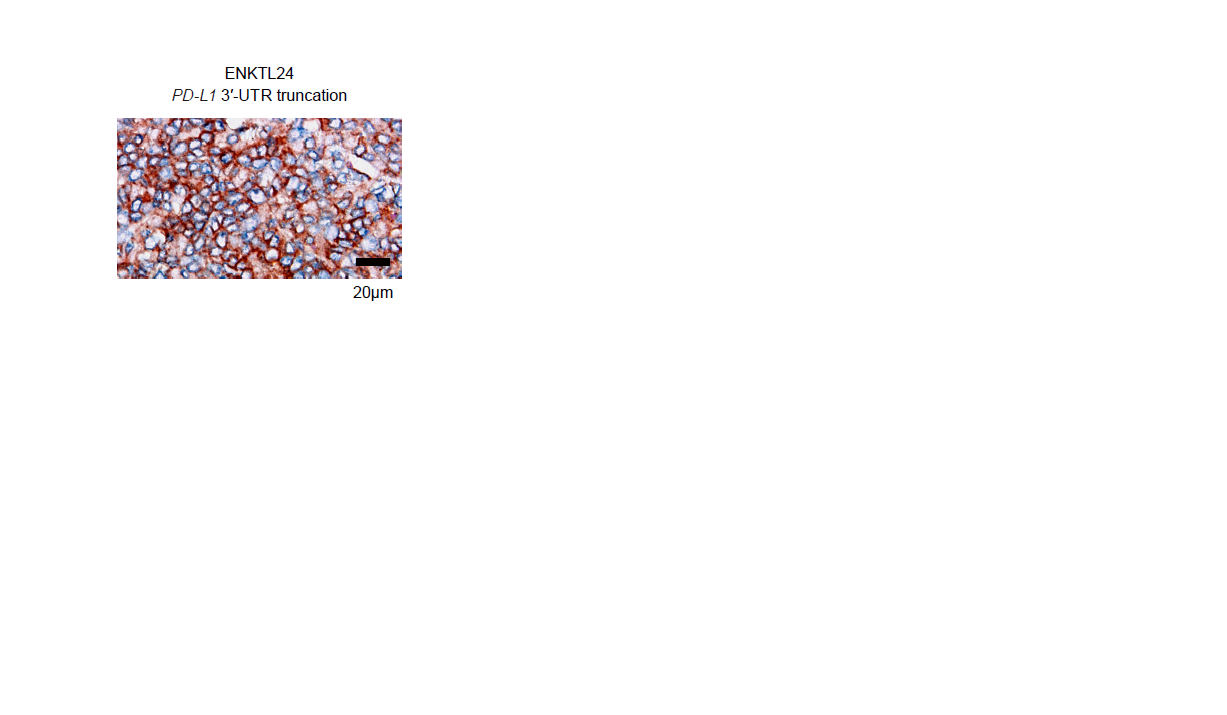
Figure S2 Double-staining of PD-L1 and CD3 in ENKTL.

Tumor cells were discriminated from surrounding immune cells by the expression of cytoplasmic CD3 (blue) and found to express PD-L1 (brown) in a representative ENKTL case.

### Figure S3 Detection of focal amplifications at 9p24.1 involving *PD-L1/PD-L2* by targeted-capture sequencing.


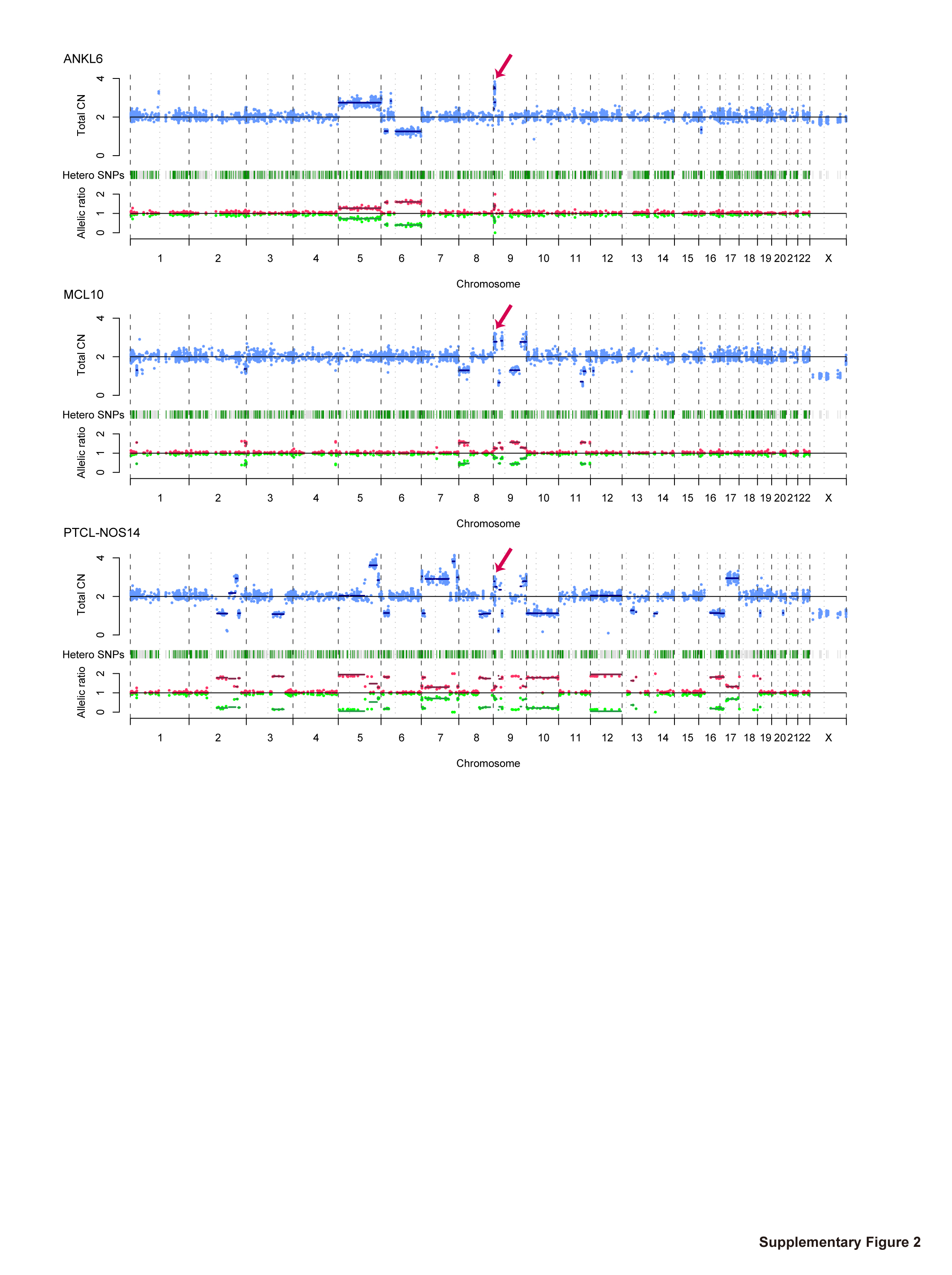


Targeted-capture sequencing data are visualized by the CNACS algorithm for representative NHL samples harboring focal amplifications involving *PD-L1/PD-L2* (indicated by red arrows). CN, copy number.

###
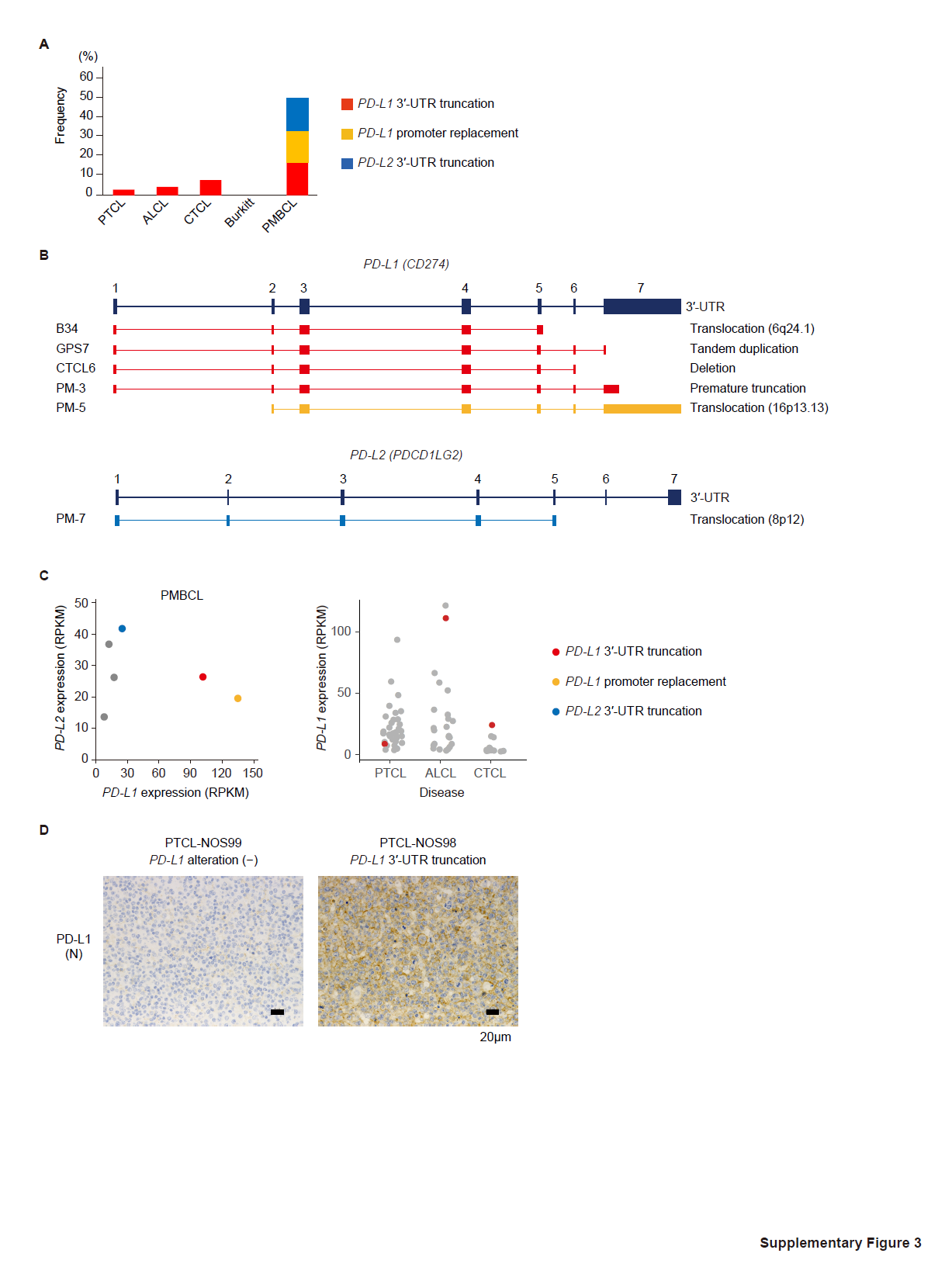
Figure S4 Aberrant transcripts involving PD-1 ligands identified by the analysis of publicly available RNA-seq data.

**A,** Frequency and type of genetic alterations involving *PD-L1* and/or *PD-L2* in each lymphoma subtype. Type of alterations is indicated by color. **B,** Different types of *PD-L1* (top) and *PD-L2* (bottom) aberrant transcripts resulting from their SVs are shown by indicated colors. **C,** *PD-L1* and/or *PD-L2* expression (RPKM) in PMBCL (left) as well as PTCL, ALCL, and CTCL (right). RPKM, reads per kilobase of exon per million mapped reads. **D,** PD-L1 IHC of PTCL-NOS cases with or without *PD-L1* genetic alterations. An antibody specifically detecting N-terminal domains of PD-L1 was used. A summary of the results is shown in **Supplementary Table S7**.

### Figure S5 Induction of *PD-L2* 3′-UTR disruption in a human cell line using the CRISPR/Cas9 system.


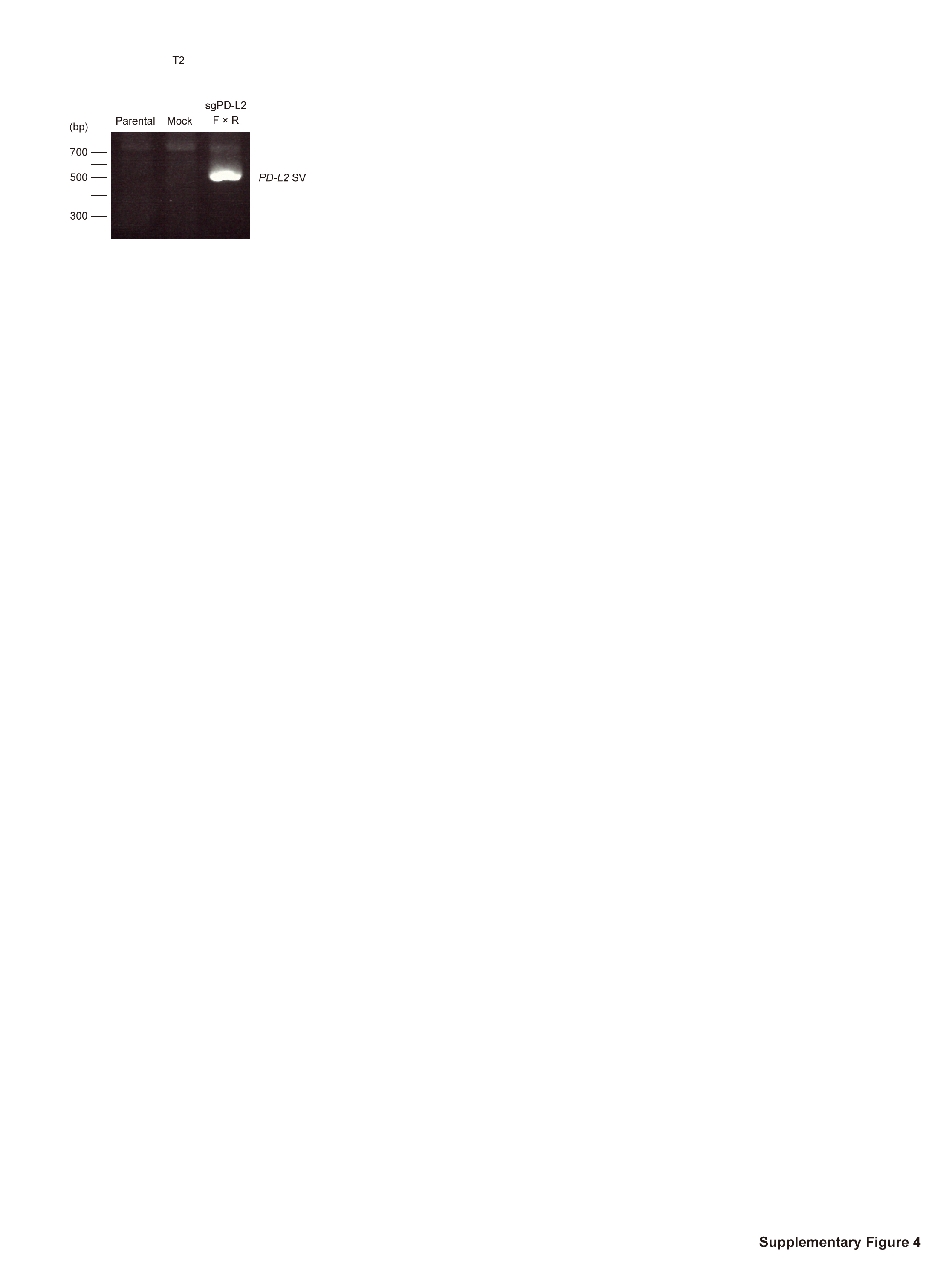


PCR detection of *PD-L2* 3′-UTR disruption in T2 cells in which Cas9 was expressed without (parental) or with no sgRNA (mock), or a pair of *PD-L2* sgRNAs.

### Figure S6 *PD-L1* SVs identified by the analysis of publicly available whole-exome sequencing data of ENKTL cases.


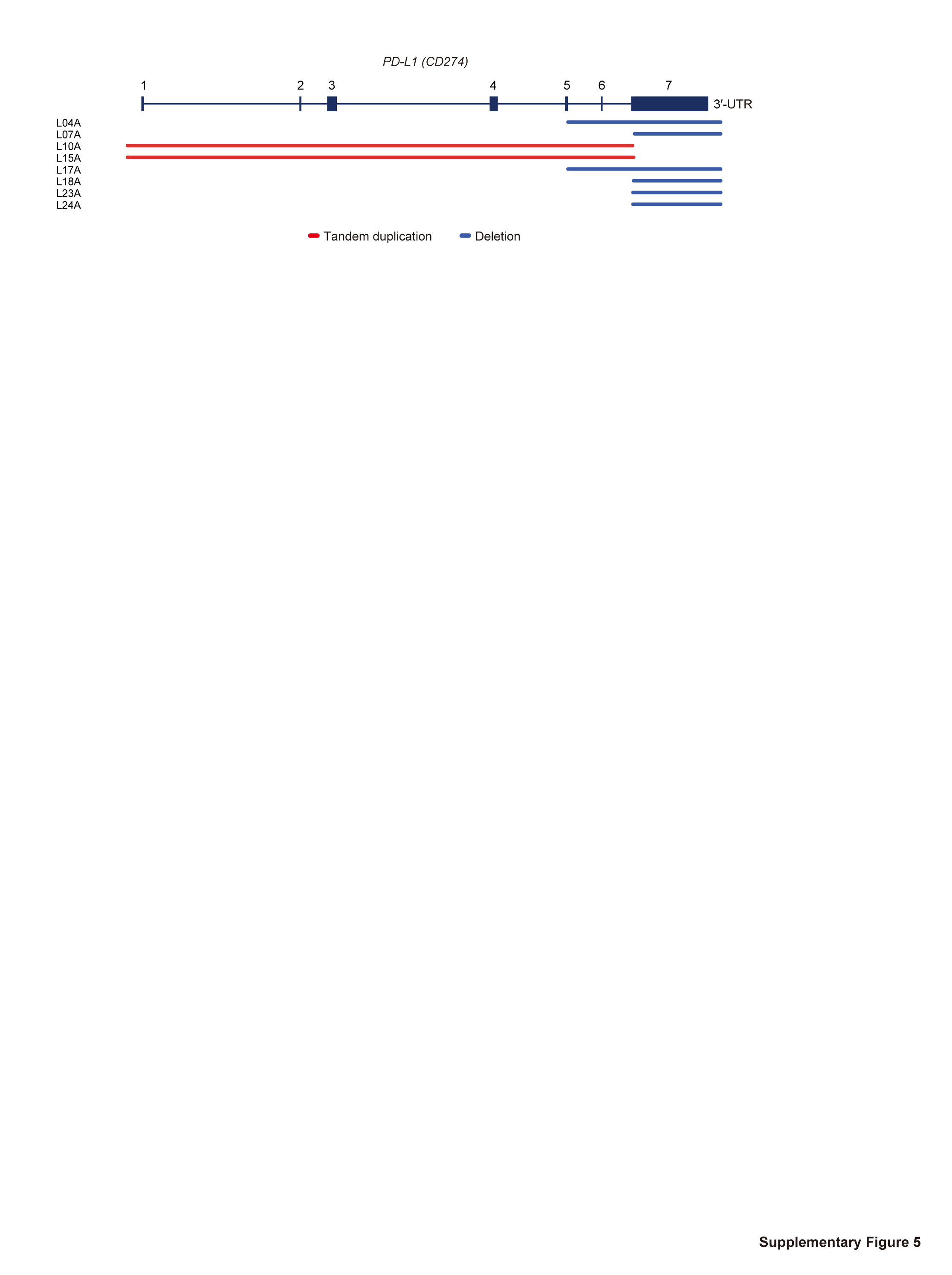


Different types of SVs affecting *PD-L1* are shown by indicated colors.

###
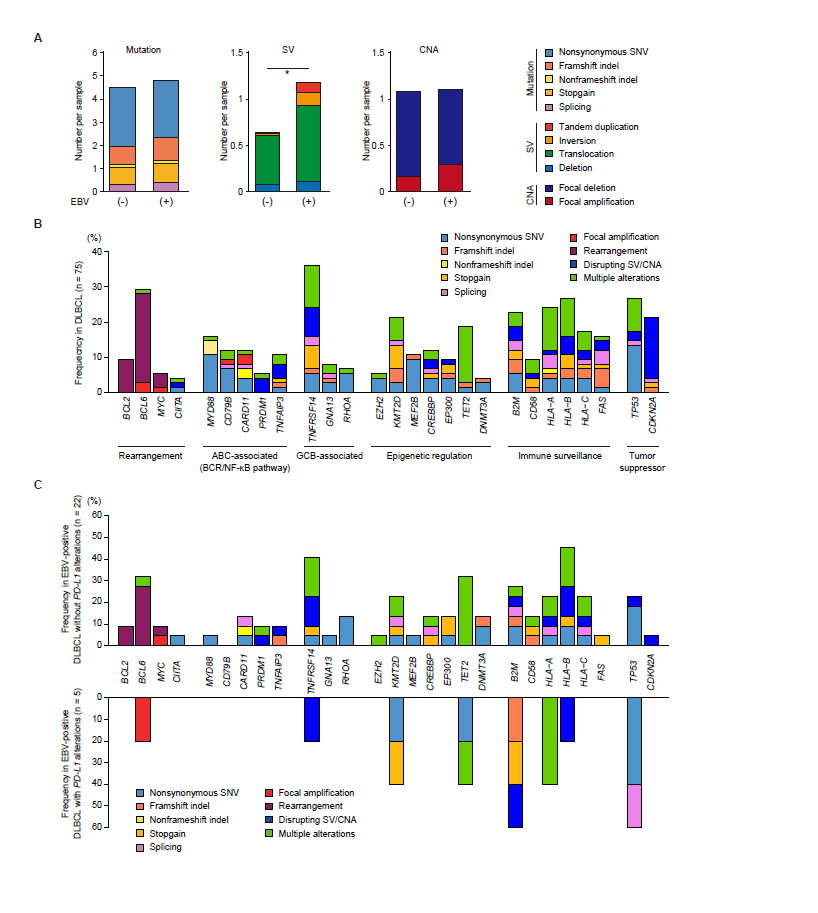
Figure S7 Genetic alterations in EBV-negative and -positive DLBCLs.

**A,** Numbers of mutations, SVs, and CNAs per sample in EBV-negative (n = 48) and -positive (n = 27) DLBCLs. Type of alterations is indicated by color. **P* < 0.05, Brunner–Munzel test. **B,** Frequency and type of somatic alterations identified by targeted-capture sequencing for lymphoma-associated genes in 75 DLBCL cases. BCR, B-cell receptor; GCB, germinal center B-cell. **C,** Frequency and type of somatic alterations in EBV-positive DLBCL cases with (n = 5) or without (n = 22) *PD-L1* genetic alterations. Type of alterations is indicated by color.
